# Lighting Up Mutation: a New Unbiased System for the Measurement of Microbial Mutation Rates

**DOI:** 10.1128/mBio.00740-19

**Published:** 2019-04-23

**Authors:** Kylie J. Boyce, Alexander Idnurm

**Affiliations:** a School of Science, RMIT University, Melbourne, Victoria, Australia; b School of BioSciences, University of Melbourne, Parkville, Victoria, Australia

**Keywords:** CRISPR-Cas9, FACS, GFP, *MSH2*, antifungal drug resistance, fluctuation test, mismatch repair

## Abstract

Although mutation drives evolution over long and short terms, measuring and comparing mutation rates accurately have been particularly difficult. This is especially true when mutations lead to an alteration in fitness. E. Shor, J. Schuyler, and D. S. Perlin (https://doi.org/10.1128/mBio.00120-19) present a new method to compare mutation rates across fungal strains and under different growth conditions: they employ the green fluorescent protein (GFP) as the reporter and count mutations using fluorescence-activated cell sorting (FACS). The estimates of mutation rates using the GFP-FACS approach are similar to those calculated with other reporters, and the method was used to assess if different alleles of the mismatch repair pathway gene *MSH2* impact the mutation rates in the human pathogen Candida glabrata. The approach could be extended to other microbes and applications, opening the way for a better understanding of how mutation rates have impacted speciation and the emergence of antimicrobial resistance.

## Abstract (precis)

Although mutation drives evolution over long and short terms, measuring and comparing mutation rates accurately have been particularly difficult. This is especially true when mutations lead to an alteration in fitness.

## COMMENTARY

The diversity of life on earth is attributed to the properties of DNA, both its remarkable stability, including during replication, and also its ability to mutate, giving variation in organisms upon which selection can act. Here lies a fine balance because mutation may have a deleterious impact to yield a less fit organism. Mutations are also the cause of many serious diseases ranging from cancer in humans to infectious diseases caused by drug-resistant microbes. Given the fundamental role of mutation in the evolution of life and its immediate relevance for human health, measuring mutation rates and comparing how they differ between different species, strains, and tissue types and under different environmental conditions are a critical aspect of biology.

The comparisons of mutation rates are challenging because the simple act of measuring the mutation rate is particularly difficult. Mutations are very rare events and have historically required a form of selection for them to be detected. Indeed, the 1969 Nobel Prize in Physiology or Medicine was awarded, in part, to Salvador Luria and Max Delbrück for their development of an assay for the quantification of mutation rates that used selection of resistance of bacteria to attack by bacteriophage as their “reporter” for mutation. The fluctuation assay, named because of the variability seen due to when in the growth of a population a mutation appears, was used to demonstrate that mutations arise in the absence of selection (1), a fundamental discovery in support for Darwin’s theory of evolution.

### Challenges and approaches of measuring mutation rates.

Little has changed since the 1940s when Luria and Delbrück used resistance to bacteriophage as their measure of mutation. The conundrum is that almost all such selection systems have a known or potential fitness change in the organism, and this therefore limits the ability to compare strains or growth conditions. For example, resistance to 5-fluoroorotic acid is commonly used to measure mutation rates in fungi, including the human-pathogenic yeast Cryptococcus neoformans. However, those mutations render C. neoformans strains sensitive to mammalian body temperature and therefore reduce virulence (2), thereby narrowing the growth parameters in which comparisons can be performed and, as a consequence, the ability to understand mutation under conditions most important for disease progression.

Furthermore, different species have different phenotypes when standard reporter genes are used or specific chemicals tested. For example, standard assays used in Saccharomyces cerevisiae based on resistance to canavanine fail to work in the very close relative Candida glabrata (3). Using drugs as the selection method for mutants has also been criticized due to the phenomenon known as adaptive evolution or postselection mutation, where mutations occur as a result of the selective pressure (4). Without a common gene target that can be used universally, it has not been possible to compare mutation rates between different species.

**FIG 1:**
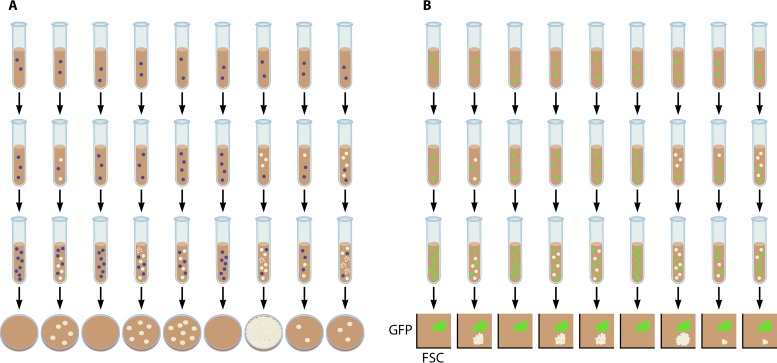
A new approach to measure mutation rates, using GFP as the reporter and FACS to detect mutations. (A) The original fluctuation test relies on culturing independent lines of a strain and then plating them onto a selective system to seek for mutations within a reporter gene or property. Disadvantages include potential fitness defects of the mutations, costs of reagents, and time in counting mutated strains. Here, blue cells indicate wild type, with white cells indicating the emergence of a mutation. The “burst” cell form represents a possible fitness penalty. (B) Shor et al. employ GFP as the reporter, which is not subject to a fitness penalty when mutated, and can count many more mutation events using cell sorting. Here, green wild-type cells are mutated to white cells, which can be detected by reduced fluorescence (GFP) by FACS (FSC, forward scatter).

Finally, the fluctuation assay itself, a gold standard for measuring mutation rates, can quickly become experimentally arduous and expensive. For instance, researchers have to consider how many parallel cultures to use, how to measure the generation time, how many plates to measure the final outcome, and then the laborious counting of colonies (Fig. 1A). The Luria and Delbrück fluctuation assay contains a number of assumptions, including that all mutants are detected, as well as that the growth rates of mutants and nonmutants are the same (5). These assumptions are unlikely to hold true for clinical isolates, which can be debilitated by mutations causing antibiotic resistance, and exhibit genetic heterogeneity and differences in the intrinsic levels of drug susceptibility (4). Alternative approaches, e.g., identifying mutations across the genome using whole-genome sequencing, hold considerable promise, yet genome sequencing and data analysis costs are likely still beyond the price range for routine measuring of mutation rates.

### A new and improved assay for measuring mutations.

Shor et al. (3) developed a new assay based on mutations in the gene encoding green fluorescent protein (GFP) and a traditional fluctuation assay counting the loss of GFP cells using fluorescence-activated cell sorting (FACS) (Fig. 1B). The team validated their assay by showing that similar estimates of mutation rates can be derived using their system as with previous reports using *CAN1* reporter assays in S. cerevisiae. This validation included using the GFP-FACS system in S. cerevisiae to compare mutation rates, in strains affected by mutation of DNA repair pathways or from exposure to mutagens. The principal advantage of this assay is that the same reporter gene can be used in a variety of different organisms so that a direct comparison of mutation rates between species and strains within a species can be performed.

The assay is not without limitations; for example, using reporter assays to measure mutation rates means that they detect only mutational events that give rise to a detectable phenotype (in this case, loss of fluorescence). This study showed that the mutation rate in a mismatch repair pathway *msh2*Δ strain was 40-fold that of the wild type; however, genome sequencing, which detects total mutations, has found that the *msh2*Δ mutant in S. cerevisiae has an >200-fold increase in mutation rate, which suggests the reporter may be missing the majority of silent mutations (6). An additional limitation of using reporter constructs to measure mutation rate is that they fail to reveal the full spectrum of possible mutations. This is especially important when analyzing the mutation rates of strains with altered genes such as *MSH2*, which generates a very specific mutational profile (6): single nucleotide deletions within runs of the same nucleotide (homopolymeric regions) represent the primary mutational event in *MSH2* mutants (6). The chosen reporter gene may not adequately reflect the primary mutational event; for example, the main cause of resistance to 5-fluoroorotic acid in C. neoformans is mutations in *URA5*, which does not contain any homopolymeric runs (7). Crucially, the mutation spectrum specific to *MSH2* mutants was recapitulated in this GFP assay, suggesting it is an appropriate reporter to assess C. glabrata clinical isolates carrying different alleles of *MSH2,* the motivation for the development of this assay by this group of researchers.

### Throwing light on a recent debate: Candida glabrata mutators.

Indeed, the incentive of the research by Shor et al. was to develop a tool for testing clinical isolates of C. glabrata that carry specific alleles of *MSH2,* to determine if they correlate with higher mutation rates that develop antifungal drug resistance. A new assay was necessary because C. glabrata is resistant to canavanine and because clinical isolates exhibit variation in their drug resistance profiles, limiting the general use of drug resistance as a reporter of mutation in fluctuation analysis. The assertion that strains with different *MSH2* alleles have increased mutation rates (or mutator phenotype), and that the presence of such alleles correlates with antifungal drug resistance, has been contentious. Although a large proportion of C. glabrata clinical isolates possess nonsynonymous variation in *MSH2* (North America, 55%; India, 69%; France, 44%; South Korea, 65%), there is not always an obvious correlation with drug resistance despite the common presence of alleles such as *V239L* in resistant isolates (65% and 69% of fluconazole-resistant isolates in North America and South Korea versus 0% and 17% in India and France, respectively) (8–11). The previous method used to assess mutation rate in C. glabrata involved reintroduction of the *MSH2* alleles into an *msh2*Δ reference strain and assessing resistance to caspofungin as the reporter (8–10). However, very different colony frequencies were observed in the *MSH2 V239L* isolates from North America and India, and some of the nonsynonymous mutations in *MSH2* complemented the *msh2*Δ strain (8, 9).

Using the new GFP C. glabrata strains and sorting for mutations with FACS, there was no difference in mutation rates between two strains with different naturally occurring alleles of *MSH2*. This suggests that the nonsynonymous mutations in *MSH2* present in clinical isolates do not result in a mutator phenotype. It is possible that the other, untested *MSH2* alleles present in the clinical isolates are mutators or that testing stress-inducing growth conditions, rather than the rich medium used, would produce different outcomes.

These results do indicate that the initial estimation of the proportion of mutators in the clinical population may be an overestimation. The hypothesis that mutators are present in fungal populations was built upon strong evidence from bacteria, where mutators are prevalent in pathogen populations (12–15). In terms of the human-pathogenic fungi, the *MSH2* homolog was identified as mutated in 2 of 11 clinical isolates of C. neoformans, both of which are mutators as shown by fluctuation analysis (7), and thus, further investigation remains warranted into how mutation rate might affect the ability of eukaryotic pathogens to cause disease and at what proportion mutators are present in clinical populations.

### Concluding remarks.

The adoption of this genetic testing system to identify mutations within an introduced target gene has enormous potential. Extending the use of GFP-FACS requires just some imagination. For instance, the doubling of chromosomes or parts of chromosomes in the human-pathogenic yeasts is one basis for fungicide resistance (16, 17), and such a system could potentially be used to measure this. That said, limitations surrounding genome integration and fungal lifestyle prohibit complete comprehensive application to fungal microorganisms. In summary, Shor et al. have developed an exciting new way to measure mutation rates in a far more precise way, providing a new tool in the arsenal of those to understand the evolution of drug resistance and to understand the forces behind the creation of diversity.
